# Comparative analysis of diagnostic techniques and treatment modalities for early-stage esophageal carcinoma: a comprehensive review

**DOI:** 10.3389/fonc.2025.1650965

**Published:** 2025-09-02

**Authors:** Jinlin Liu, Min Zhang, Min Zhu, Simin Tan, Xuefeng Luo, Jia Liu, Hai Zeng

**Affiliations:** ^1^ Department of Clinical Medicine, The First Affiliated Hospital of Yangtze University, Jingzhou, Hubei, China; ^2^ Department of Oncology, The First Affiliated Hospital of Yangtze University, Jingzhou, Hubei, China

**Keywords:** early esophageal cancer, endoscopic resection, surgery, definitive chemoradiotherapy, treatment advancements

## Abstract

Esophageal cancer (EC) is a significant global health burden, early disease management has witnessed substantial advancements in recent years. While surgical resection remains the cornerstone, emerging organ-preserving methods-including endoscopic resection (ER), definitive chemoradiotherapy (dCRT), and adjuvant therapies-are becoming viable alternatives for pT1a-m3/pT1b EC. This review critically evaluates contemporary diagnostic methods and emphasizes the critical role of advanced endoscopic techniques, such as Narrowband Imaging Magnifying Endoscopy (ME-NBI) in overcoming the challenge of sufficient recording for accurate TN staging. We systematically evaluated the treatment options for T1 lesions and compared the differences in survival outcomes, complications, and quality of life impact between ER, surgery, and chemoradiotherapy (CRT). Particular attention is given to the risk stratification of lymph node metastasis (LNM) and its impact on treatment selection. This review establishes an evidence-based risk stratification framework for LNM, informing clinical decision-making. ER is recommended for high-risk patients, while ER-CRT is an effective option for patients with lower recurrence risk. ER shows non-inferior survival to surgery with better functional outcomes (5-yr OS 90% vs 87%), while CRT provides organ preservation at higher stenosis risk (33%), per JCOG0502 and NCCN guidelines. By integrating data from key trials and current guidelines, this work clarifies ongoing controversies while highlighting emerging directions, including artificial intelligence(AI) enhanced endoscopic diagnosis and optimized adjuvant therapy. This analysis provides a comprehensive, evidence-based perspective for the rapidly developing field of gastrointestinal oncology.

## 1 Introduction

EC is one of the most common malignant tumors worldwide and the sixth leading cause of cancer death (1). Geographic disparities are striking, with age-standardized incidence rates (ASIR) varying 20-fold between high-risk regions (e.g., Eastern Asia: 16.7/100,000) and low-risk areas (e.g., Western Europe: 0.8/100,000) (1, 2). This reflects distinct etiological patterns: tobacco/alcohol dominate in Europe/North America (attributable fraction 85-90%), while thermal injury from hot beverages and dietary nitrosamines contribute substantially in Asia’s ‘ESCC belt’ (Henan, China: ASIR 112.3/100,000) (3, 4). With the extensive application of esophagogastroduodenoscopy in screening, the detection rate of early stage EC is steadily rising. In 2015, in Japan, the proportion of newly diagnosed EC patients at clinical stage IA (AJCC 7th edition) reached 33.4% (5). The 5-year survival rate was 73% - 86% (6). Global data reveal significant differences in survival metrics for stage I ESCC: while 5-year overall survival (OS) ranges from 61.9% to 86% (incorporating all-cause mortality) (2, 7), cancer-specific survival (CSS) reaches 90-97.1% when assessing only disease-related deaths (8). This 25–35 percentage point discrepancy primarily reflects competing risks from comorbidities in this predominantly elderly population (9).

Although there are increasingly more staging diagnosis and treatment methods for early EC, most of these studies are single center retrospective studies. While some studies have demonstrated that adjuvant esophagectomy is superior to adjuvant CRT in high-risk patients, the long-term prognosis and quality-of-life impact of different treatment modalities across various risk stratifications still require further investigation. Therefore, it is necessary to analyze the cumulative data of existing related studies. In this review, we will summarize and critically discuss various staging methods and treatment options for early EC.

## 2 Diagnosis and risk stratification

### 2.1 Definition of early-stage esophageal cancer

**Figure 1:**
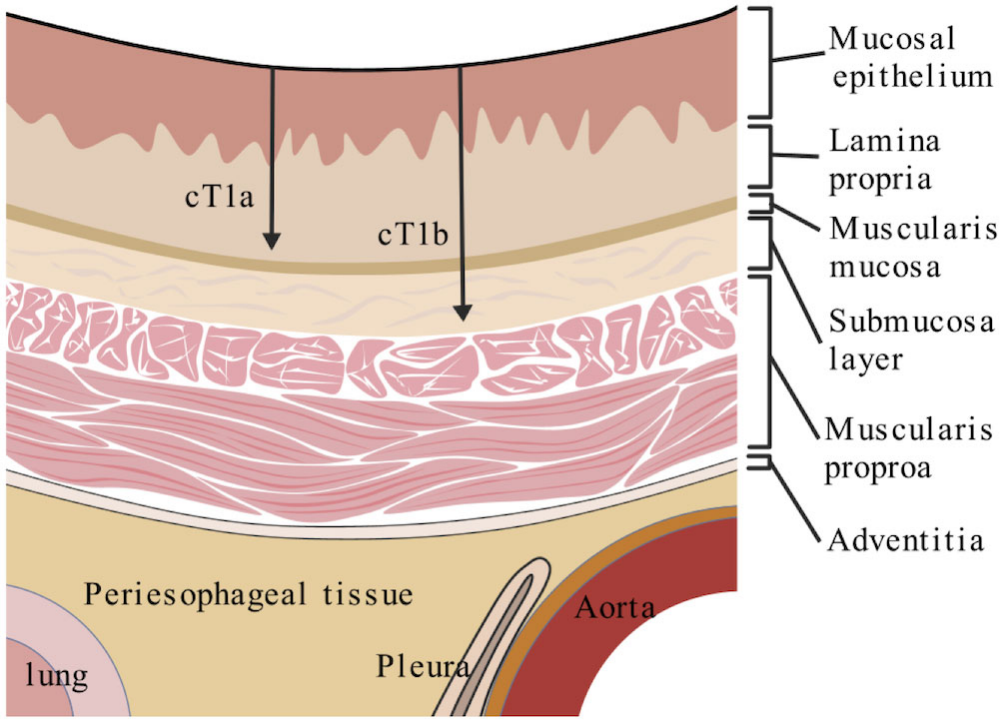
Schematic diagram of the esophageal wall layers, including mucosal epithelium (EP), lamina propria (LP), muscularis mucosa (MM), submucosal layer (SM), and muscularis propria (MP).

The esophageal mucosal structure consists of the mucosal epithelium, lamina propria, muscularis mucosae(MM),submucosa, muscularis propria, and the esophageal epithelium (**Figure 1**). Depending on the surgically resected specimen, the submucosa is divided into three layers, namely SM1: the upper third of the submucosa; SM2: the middle third of the submucosa, and SM3: the lower third of the submucosa. In endoscopically resected specimens, SM1 is defined as infiltration from the MM to 200 micrometers, and deeper levels of infiltration are categorized as SM2 and SM3 (10).According to the American Joint Committee on Cancer(AJCC), 8 edition (11), primary EC with tumor cells are limited to the epithelium and are defined as Tis. The tumor is limited to the mucosal epithelial layer, lamina propria and MM, defined as T1a, while the tumor is located in the submucosal layer, defined as T1b. EC with tumor invasion depth of Tis, T1a and T1b, but without LNM, is defined as early EC (12). However, we need to note that in the 8th edition of AJCC staging, for EC, the clinical staging of T1N0M0, and T1N1M0 belongs to stage I (11).This manuscript mainly discusses the diagnosis and treatment of EC with stage T1N0M0.

### 2.2 Early detection and diagnostic accuracy of early-stage esophageal cancer

Histopathology is the gold standard for diagnosing early stages of EC; however, accurate clinical staging before treatment is crucial for selecting appropriate therapeutic strategies. The consistency between clinical judgment of tumor infiltration depth and pathological diagnosis of infiltration depth in EC is not high. Even if clinically diagnosed as mucosal muscle layer or SM1 stage before treatment, 27.4%-55.2% of patients are pathologically confirmed to be limited to the epithelial layer or mucosal lamina propria. On the contrary, 15.5%-27.9% of pathological cases were confirmed to have deep infiltration in SM2 stage, and clinical staging usually indicates deeper infiltration (13). White light endoscopy (WLE) demonstrates characteristic but often subtle findings in early EC, including patchy erythematous or pale mucosal discoloration, slightly elevated or depressed lesions with irregular surfaces, erosion or nodular changes, and blurred or absent submucosal vascular patterns (14). However, the diagnostic sensitivity of WLE remains limited, with an approximate 7.9% missed diagnosis rate (15) primarily attributable to two key factors: hemoglobin absorption in tumor stroma that obscures underlying vascular networks, and the minimal morphological alterations typically present in flat or superficial neoplastic lesions (16). These limitations highlight the need for more advanced diagnostic techniques to improve the accuracy of early-stage EC detection.

Lugol’s chromoendoscopy operates through glycogen depletion in dysplastic epithelium (16), creating characteristic unstained areas with demonstrated sensitivity of 80.5% (**Table 1**), specificity of 94.3%, and overall accuracy of 90.5% (17). However, this method presents several limitations: contraindications in patients with hyperthyroidism or iodine allergy (18), decreased specificity (66.0%) due to inflammatory false positives (19), and practical disadvantages including prolonged examination duration (5.15 minutes) and increased biopsy requirements (41.11% of cases) (20).

**Table 1:** Diagnostic performance of endoscopic techniques for early EC.

Technique	Sensitivity (95% CI), %	Specificity (95% CI), %	Key advantages	Major limitations	Clinical use case	References
WLE	NR	NR	Widely available; low cost	High missed diagnosis rate (7.9%)	Initial screening despite low sensitivity	(14–16)
Lugol’s chromoendoscopy	80.5 (75.2–85.0)	94.3^*^ (91.1–96.5)	High accuracy for mucosal survey	Iodine contraindications, long exam time (5.15 min)	High-risk population screening (JES Grade A recommendation)	(10, 16–20)
ME-NBI	78.2 (B1 type)	NR	Real-time vascular assessment	Operator-dependent, low specificity for B2 lesions	Intraprocedural depth staging (NCCN Category 2A)	(21, 31, 62)
AI-aided ME-NBI	94.7 (90.1–97.3)	91.2 (86.5–94.5)	Standardized interpretation	Requires validation in diverse populations	Tertiary center quality control	(23, 27, 41)

WLE, white-light endoscopy; NR, not reported; ME-NBI, magnifying endoscopy with narrow-band imaging; AI-aided ME-NBI, Artificial Intelligence-aided Magnifying Endoscopy with Narrow-Band Imaging; CI, confidence interval. Data derived from comparative studies cited in brackets. *Lugol’s specificity (94.3%) reflects (17), adjusted for inflammatory false positives in (19).

Sensitivity/specificity data are derived from comparative studies cited in brackets.

AI-aided systems show superior sensitivity but depend on hardware/training datasets.

Data derived from comparative studies cited in brackets.

Narrow-Band Imaging(NBI) employs optical filters (415nm and 540nm wavelengths) to enhance microvascular contrast (21), achieving comparable sensitivity (100%) to Lugol’s while demonstrating superior specificity (79.9% versus 66.0%) (19). The technique exhibits a positive predictive value of 7.69% (versus 8.11% for Lugol’s) and offers significant procedural advantages, including reduced examination time (3.5 minutes) and lower biopsy frequency (12.75% of cases) (20, 22). While NBI’s operational advantages make it preferable for routine practice, Lugol’s retains value for pan-mucosal screening in high-risk populations. Nevertheless, both techniques remain operator-dependent-a limitation now being addressed by AI technologies.

Recent advances in AI, has demonstrated potential to mitigate operator-dependent limitations in endoscopic diagnosis. Deep learning algorithms, particularly convolutional neural networks (CNNs), achieving real-time analysis of microvascular patterns in NBI with reported accuracy exceeding 90% for early EC detection (23). Multicenter validations show that AI-assisted systems can reduce missed diagnosis rates by 40–50% compared to conventional white-light endoscopy (13), primarily through standardized interpretation of subtle mucosal changes. However, the clinical integration of these technologies requires further validation in prospective trials to address heterogeneity in lesion morphology across populations.

AI-driven quantitative histomorphometric analysis shows promise in refining risk stratification. Deep learning algorithms classifying ME-NBI patterns (e.g., B2/B3 subtypes) exhibit strong concordance with histopathologic invasion depth (κ=0.82 for SM2+ lesions vs. κ=0.54 for endoscopic visual assessment) (24). Furthermore, predictive models incorporating vascular density metrics and lymphovascular invasion(LVI) status demonstrate superior discriminative capacity for high-risk T1b-SM2/3 lesions (AUC 0.91, 95% CI 0.87–0.94) (25). These tools could potentially guide personalized therapeutic decision-making.

Although AI models perform well on certain specific tasks, their generalization ability still needs further validation. For example, studies have shown that AI systems perform poorly in predicting B2/B3 type blood vessels, possibly due to the wide explanatory range of B2 type blood vessels and the higher incidence of B3 type blood vessels being misdiagnosed as B2 type blood vessels (26). In addition, there are significant differences in the operational skills of different endoscopists, which may lead to significant differences in the performance of AI models among different doctors.

The application of AI technology in real-time endoscopic diagnosis is still in its early stages. Although AI assisted systems can improve the detection rate of early EC, their accuracy and efficiency in real-time diagnosis still need further research and validation (27). For example, some studies have pointed out that the performance of AI systems in real-time endoscopic examinations has not yet reached the level of endoscopists, and more clinical trials are needed to evaluate their efficiency in different medical environments (28). The edge lesions of EC are often difficult to accurately identify through traditional endoscopic examination, and the application of AI technology in this field still faces challenges. For example, AI systems may not be as accurate as endoscopists in identifying edge lesions that are difficult to determine (29). In addition, the lack of genetic diagnostic markers also limits the application of AI in pathological diagnosis (29).

### 2.3 Diagnosis of invasive depth in early esophageal cancer

The Japan Esophageal Society (JES) has established a consensus-based ME-NBI classification system that standardizes depth prediction for superficial ESCC (30). This system categorizes lesions according to distinct microvascular patterns (31)(The classification performance of ME-NBI is shown in **Table 2**):

**Table 2:** Diagnostic performance of magnifying endoscopy with narrow-band imaging (ME-NBI) for predicting invasion depth in early esophageal squamous cell carcinoma.

ME-NBI type	Microvascular pattern	Corresponding depth	Sensitivity (95% CI), %	Specificity (95% CI), %	Clinical implication
B1 (31)	Abnormal intraepithelial looped vessels	Epithelium (m1) or lamina propria (m2)	71.4 (51.1–86.0)	100 (89.6–100)	Curative endoscopic resection feasible
B2 (21)	Elongated, non-looping microvessels	Muscularis mucosae (m3) or SM1 (≤200 μm)	94.4 (70.6–99.7)	73.1 (36.3–72.2)	Requires EUS confirmation due to overestimation risk of m3/SM1 lesions
B3 (30)	Irregular, dilated vessels (>3× B2)	Deep submucosa (SM2, >200 μm)	75.0 (52.9–89.4)	97.8 (87.0–99.9)	Surgical resection recommended

ME-NBI, Magnifying Endoscopy with Narrow-Band Imaging; CI, Confidence Interval. AUC: Area Under the Curve.

Interobserver agreement κ=0.87 (95% CI: 0.76–0.95) derived from (31).B2 lesions require EUS confirmation due to overestimation risk; B3 lesions warrant surgical resection.

Type B1: Characterized by abnormal intraepithelial microvessels with preserved loop-like structures, corresponding to tumors confined to the epithelium (m1) or lamina propria (m2). It demonstrates moderate sensitivity (71.4%, 95% CI: 51.1–86.0) but perfect specificity (100%; 95% CI: 89.6–100), making it highly reliable for excluding deeper submucosal invasion (31).

Type B2: Defined by elongated, non-looping microvessels, indicative of invasion into the muscularis mucosa (m3) or superficial submucosa (sm1; ≤200 μm). While it shows high sensitivity (94.4%; 95% CI: 70.6–99.7), its specificity is suboptimal (73.1%; 95% CI: 36.3–72.2), leading to potential overestimation of m3/sm1 lesions (31).

Type B3: Marked by irregular, dilated vessels exceeding threefold the caliber of B2 vessels, correlating with deep submucosal invasion (sm2; >200 μm). It exhibits moderate sensitivity (75.0%; 95% CI: 52.9–89.4) and near-perfect specificity (97.8%; 95% CI: 87.0–99.9), critical for guiding surgical resection (31).

The overall diagnostic accuracy of this system is 78.6%, with excellent interobserver agreement (κ=0.87; 95% CI: 0.76–0.95). However, limitations persist in differentiating B2 lesions, necessitating adjunctive techniques such as endoscopic ultrasonography for precise staging.

The operator-dependency of endoscopic techniques poses significant challenges, particularly in differentiating B2/B3 microvascular patterns (inter-observer variability κ=0.54-0.67) (31). Structured training programs like the Japan Endoscopy Society’s 50-case certification system have demonstrated 35% improvement in novice endoscopists’ accuracy for depth prediction (13). Emerging AI solutions show particular promise: real-time systems (e.g., CAD-EYE) reduce interpretation variability by standardizing microvascular assessment, achieving 89.4% concordance with expert review in multicenter trials (95%CI 86.2-92.1) (26). However, current models require optimization for lesions with atypical vascular patterns (e.g., type B2v with sparse vasculature, where accuracy drops to 72%) (29).

Endoscopic ultrasound (EUS) plays a critical role in T-staging of early EC, demonstrating 53.9% sensitivity and 85.0% specificity in differentiating T1b (submucosal) from T1a (mucosal) lesions, with an overall T-stage concordance rate of 75.5% compared to histopathology (32). However, precise discrimination between SM1 (superficial submucosal) and SM2/SM3 (deeper submucosal) invasion remains challenging due to limited resolution for subtle submucosal layer differentiation (32).

For nodal (N) staging, EUS relies on morphological criteria such as lymph node size, shape, and echogenicity; non-diagnostic lymph nodes (lacking definitive malignant features) were observed in 50% of cases, and none met established criteria for metastasis (32, 33). Despite its utility, EUS exhibits notable limitations: Understaging occurred in 46.2% of T1b lesions (misclassified as T1a), while overstaging was observed in 14.3% of T1a lesions (erroneously labeled as T1b). Key contributing factors include (34, 35):(1) Technical dependence on initial endoscopic screening to localize suspicious lesions; specifically, EUS requires identification of the suspicious lesion via standard endoscopy first, followed by positioning the ultrasound probe in optimal contact with the lesion to accurately assess its depth of invasion. Achieving and maintaining this optimal contact is particularly challenging in anatomically complex regions (e.g., proximal esophagus); (2) Artifacts from post-biopsy inflammation or ulceration, which alters tissue echogenicity; (3) Operator-dependent interpretation of submucosal irregularities and lymph node morphology.

The combination of ME-NBI and EUS synergistically enhances diagnostic performance, achieving pooled sensitivity of 0.947 (95% CI: 0.901–0.975) and specificity of 0.894 (95% CI: 0.847–0.931) for early EC detection, with an AUC of 0.97 (36). For invasion depth staging, the combined approach yields sensitivity of 0.791 (95% CI: 0.674–0.881) and specificity of 0.943 (95% CI: 0.906–0.968), supported by an AUC of 0.95 (36). While ME-NBI optimizes superficial lesion characterization and EUS improves deep-layer assessment, persistent challenges include operator dependency, SM1/SM2 differentiation, and regional validation biases (predominantly Chinese cohorts) (37). Future studies should prioritize large-scale, multicenter validations to refine staging protocols and address technical limitations (38).

**Table 3** summarizes the diagnostic performance of major endoscopic modalities for early EC, highlighting the diagnostic accuracy of techniques for clinical T staging in early EC.

**Table 3:** Diagnostic accuracy of endoscopic techniques for clinical T staging in early EC.

Technique	Target T stage	Sensitivity (95% CI), %	Specificity (95% CI), %	Advantages	Limitations
EUS	T1a vs. T1b	53.9 (42.1–65.2) (32)	85.0 (76.3–91.0) (32)	Evaluates submucosal invasion Detects suspicious lymph nodes	Overstages 14.3% of T1a as T1b Understages 46.2% of T1b as T1a
ME-NBI + EUS	T1a vs. T1b	94.7 (90.1–97.3) (36)	89.4 (84.7–92.8) (36)	Synergistic improvement in accuracy	Requires expert operators
Post-ESD Pathology	Final T staging	100 (Gold standard) (11, 56)	100 (Gold standard) (11, 56)	Definitive for T1a/T1b classification	Only applicable after resection

EUS, Endoscopic Ultrasound; ME-NBI, Magnifying Endoscopy with Narrow-Band Imaging; ESD, endoscopic submucosal dissection. CI: Confidence Interval; AUC: Area Under the Curve. EUS sensitivity for T1a vs. T1b differentiation varies by operator experience (range: 45-65%).

### 2.4 Non-endoscopic and advanced detection methods

Non-endoscopic screening methods are transforming early EC detection through minimally invasive techniques. Sponge cytology has proven safe and feasible for squamous neoplasia screening, while emerging liquid biopsy approaches demonstrate superior diagnostic performance. Salivary miRNA-21 exhibits high accuracy for T1 EC detection (82.3% sensitivity, 91.6% specificity; AUC 0.89), significantly outperforming conventional serum biomarkers (AUC 0.72) (39). Similarly, plasma 5hmC signatures detect stage I EC with 73.5% sensitivity at 90% specificity (40), and cfDNA methylation markers provide complementary molecular insights.

The integration of these multi-omics approaches with AI-assisted risk stratification tools (41) and clinical parameters enables comprehensive pre-endoscopic screening. Notably, combining liquid biomarkers with AI-based image analysis may further enhance detection efficacy. Machine learning models incorporating salivary miRNA profiles and endoscopic NBI features demonstrate additive effects, achieving 94.7% sensitivity for T1a tumors-surpassing either modality alone (39). Such multimodal strategies could improve screening in high-risk populations by mitigating the limitations of non-specific biomarkers.

Despite their diagnostic potential, pre-analytical variables (e.g., sample collection timing, RNA stabilization methods) and lack of FDA/CE-approved kits currently limit routine clinical implementation (39). And multicenter prospective studies remain imperative to validate clinical utility and standardize implementation protocols prior to routine adoption.

### 2.5 Risk stratification in early esophageal cancer

Contemporary management of early EC requires precise risk stratification based on depth of invasion and lymphovascular status. **Table 4** summarizes the risk stratification criteria and corresponding treatment recommendations, along with the level of evidence supporting each recommendation.

**Table 4:** Risk stratification by invasion depth and lymph node metastasis in early EC.

Invasion dSepth	Pathologic criteria	LNM rate (%)	5-Year OS (%)	Recommended treatment	Evidence level
Low-risk (pT1a-m1/m2)	Epithelium/lamina propria	0.4	92.3	ESD alone	JCOG0508 (8) JES Guideline (57)
Intermediate-risk (pT1a-m3/LVI+)	Muscularis mucosae/LVI+	5.3–30.8	71.1	ESD + adjuvant CRT (50.4 Gy)	Kawaguchi et al. (55)
High-risk (pT1b-SM2/3)	Deep submucosa	36.2	54.6	Esophagectomy + D2 lymphadenectomy	NCCN v2.2023 (62), Hölscher et al. Ann Surg 2011 (42)

LNM, lymph node metastasis; LVI, lymphovascular invasion; ESD, endoscopic submucosal dissection; CRT,. Chemoradiotherapy.

## 3 Treatments for early-stage esophageal cancer

Current treatment strategies for early EC are stratified by invasion depth and LNM risk, with key outcomes summarized in **Table 4**. The following sections detail each modality.

### 3.1 Lymph node metastasis in early esophageal cancer

LNM status is the most critical prognostic factor in early-stage EC and a major factor in determining treatment options, with pN0 patients show significantly better 5-year survival than pN+ cases (82% vs. 45%, respectively) (42). Risk stratification should integrate tumor invasion depth, LVI, and anatomical location. Current evidence demonstrates a depth-dependent metastatic gradient: mucosal lesions (MM1, MM2, MM3) exhibit LNM rates of 0%, 1.5–3.7%, and 5.3–30.8%, respectively (25), while submucosal invasion (SM1, SM2, SM3) escalates to 13%, 19%, and 56% (42). Yang et al. further validated this pattern in a multicenter study of 241 pT1N+ patients, where pT1b cases (89.2%)—particularly SM3 (54.4%)—demonstrated high metastatic propensity. Notably, even in the absence of LVI, SM2/SM3 tumors retain substantial LNM risk (28.6–37.9%) (43), and Nerve Infiltration (NI) independently predicts poor prognosis (P=0.036 (44). Survival analyses confirm that SM1/SM2 lesions achieve a combined 5-year survival rate of 86%, significantly higher than SM3 (46%, P = 0.008) (42), while adequate lymphadenectomy (>28 nodes) improves outcomes (P = 0.026). Anatomically, the recurrent laryngeal nerve (RLN) lymph nodes (stations 106recR/L) exhibit the highest metastatic rates (35.8%/25.6%), with upper thoracic tumors showing 106recR involvement in 50% of cases (44). Thus, diagnostic strategies should incorporate ME-NBI and EUS for precise staging. Current treatment algorithms follow risk-adapted principles: low-risk (MM1–MM2) lesions warrant ER; intermediate-risk (MM3–SM1) cases require individualized adjuvant therapy; and high-risk (SM2–SM3 + LVI) disease necessitates radical esophagectomy with systematic lymphadenectomy, including RLN nodal basins. Prospective studies are needed to optimize adjuvant therapy for pT1N+ patients, as current evidence shows no survival difference between chemotherapy and CRT (P = 0.093) (45). Given the prognostic significance of LNM, the following sections detail treatment modalities stratified by the above risk criteria.

### 3.2 Endoscopic submucosal dissection with adjuvant therapy for high-risk lesions

The selection of ER modalities must be guided by rigorous risk stratification as detailed in Section 3.1. Recent advancements in endoscopic techniques, including endoscopic mucosal resection (EMR), ESD, radiofrequency ablation (RFA), and photodynamic therapy (PDT), have significantly improved the management of early EC. EMR is indicated for lesions ≤20 mm with low risk of submucosal invasion (SM1), as it allows en bloc resection in select cases and provides sufficient tissue for histopathological evaluation of invasion depth and differentiation (46, 47). However, piecemeal resection for larger lesions (>20 mm) results in fragmented specimens, limiting accurate margin assessment and leading to higher recurrence rates (10–12%) (48). Prophylactic ablation of residual mucosa post-EMR may reduce recurrence in extensive lesions. While EMR is associated with low perforation rates and minimal intraprocedural complications, delayed bleeding occurs in 6.2% of cases (48, 49). A meta-analysis of 1,081 patients demonstrated superior curative resection rates for ESD (92%) compared to EMR (53%), with recurrence rates of 0.3% versus 12%, respectively (49). ESD is particularly advantageous for bulky lesions, intramucosal carcinoma, or those with superficial SMI (<500 μm) (50). However, ESD requires advanced technical expertise and is associated with higher risks of perforation (4–5%) and post-procedural stricture (11.6%) (48). A prospective randomized trial reported en bloc resection rates of 100% for ESD versus 15% for EMR in early esophageal neoplasia (51). Lesion selection should prioritize tumor size, SMI risk, and histology. EMR remains suitable for small, low-risk lesions, while ESD is preferred for lesions ≥20 mm, those with suspected SMI, or incomplete prior resections.

While EMR is initially less expensive than ESD due to shorter procedure time and lower technical demands, long-term cost-effectiveness must account for recurrence rates and the need for repeat procedures, and even repeated non curative surgeries will increase cumulative costs (48). A meta-analysis by Guo et al. demonstrated that ESD had significantly lower recurrence rates (0.3% vs. 12%) compared to EMR, reducing the need for additional interventions and associated costs. However, ESD requires specialized training and has higher upfront costs, including equipment and longer procedural time (average 90–120 minutes vs. 30–60 minutes for EMR) (49). A study by Yang et al. further quantified the economic impact, showing that the cumulative cost of EMR over 5 years (including repeat procedures for recurrence) exceeded that of ESD by approximately 20%, primarily due to higher recurrence-related expenses (e.g., surveillance endoscopies, salvage therapies) (50). Additionally, ESD’s superior en bloc resection rate (92% vs. 53%) reduces the risk of incomplete resection, which may necessitate costly adjuvant therapies or surgery (49, 51).

Prophylactic measures post-EMR (e.g., radiofrequency ablation of residual mucosa) can mitigate recurrence but add to procedural costs. In contrast, ESD’s higher initial cost may be offset by its durability, particularly for lesions >20 mm or with suspected submucosal invasion (48). These findings underscore the importance of lesion-specific selection to optimize both clinical outcomes and cost efficiency.

RFA serves as a non-resectional therapy for extensive superficial lesions (e.g., Barrett’s esophagus with high-grade dysplasia), demonstrating low recurrence rates and minimal complications, though its efficacy is confined to mucosal lesions without addressing submucosal invasion (52).

PDT, utilizing photosensitizers like porfimer sodium or talaporfin sodium, achieves high complete response rates (87–88.5%) in early ESCC and salvage therapy for local failure post-CRT, with reduced phototoxicity in second-generation protocols (53). While PDT preserves organ function and induces immunogenic tumor death, its application is limited by stricture risk (25% with first-generation agents), stringent lesion criteria (diameter ≤2 cm, no deep submucosal invasion), and competition from ESD/RFA. Lesion selection should integrate tumor size, depth, and patient comorbidities, positioning PDT as a salvage or alternative option for non-surgical candidates or multifocal lesions unamenable to resectional therapies (54).

The JCOG 0508 trial (8), a prospective study of 177 patients with clinical stage T1aN0M0 or T1b-SM1/2N0M0 ESCC treated with ESD followed by risk-adapted adjuvant therapy, demonstrated comparable oncologic outcomes to surgical resection. The study cohort was stratified into three groups based on pathological findings: Group A (pT1a with negative margins and no LVI) underwent observation; Group B (pT1a with LVI or pT1b with negative margins) received prophylactic CRT; and Group C (positive/uncertain margins) underwent curative-intent CRT. The 5-year progression free survival rates for Group B and all enrolled patients were 86.2% and 87.5%, respectively. The overall 5-year survival rates for Group B and all enrolled patients were 89.7% and 90.9%, respectively. The 5-year survival rate of Group B is similar to that of stage 1 esophageal squamous cell carcinoma undergoing radical surgery (7). Of course, we also need to note that this is not a randomized controlled study. In addition, within a median follow-up of 81.5 months, the proportion of patients with local lymph node failure as the first recurrence site was 0%, 10.84%, and 15.38% in groups A, B, and C. One patient in Group B developed grade 3 esophageal stenosis and was unable to complete adjuvant chemotherapy. Adverse events of grade 3 or higher caused by radiotherapy and chemotherapy include neutropenia (22.9%), hyponatremia (7.3%), esophagitis (4.2%), and anorexia (7.3%).

For high-risk lesions (pT1a-MM3/LVI+ or pT1b-SM1), adjuvant therapy post-ESD demonstrates significant benefits. The JCOG0508 trial (8) reported 3-year locoregional control rates of 100% with adjuvant radiotherapy versus 57.8% without (p=0.02), while Kawaguchi et al. (55) showed ESD-CRT achieved superior 3-year OS compared to dCRT alone (90.0% vs 63.2%). These outcomes must be balanced against CRT-associated toxicities (grade ≥3 esophagitis: 4.2% (8). Absolute indications include clinical/pathological Tis or T1a lesions without evidence of LVI or poor differentiation, while relative indications encompass pT1a-MM3 lesions (particularly with LVI) and pT1b-SM1 lesions (demonstrating a 13.2% recurrence risk in the absence of LVI) (56–58). Contraindications comprise deep submucosal invasion (SM2/SM3), presence of LVI, or poorly differentiated histology (57).

Lesions pathologically confined to the lamina propria or MM (pT1a) or superficial submucosa (pT1b) can be endoscopically resected in the absence of evidence of LNM, vascular-lymphovascular infiltration, or hypo-differentiation (59). For patients with limited early lesions (Tis and T1a ≤2 cm, highly or moderately differentiated carcinoma), endoscopic treatment is considered “preferred” because of the lower risk of LNM, local or distant recurrence, and death from EC after endoscopic treatment (60). ER is encouraged for small nodal lesions ≤2 cm because it provides a more accurate depth of infiltration than EUS (61).

pT1a-MM1 and pT1a-MM2 have a very low risk of lymph node recurrence and have been determined to be curatively resected by ER, so no additional treatment is needed (62). pT1a-MM3 has a risk of metastasis and recurrence, especially if accompanied by LVI, and requires additional treatment (57). Surgical resection or CRT is strongly recommended as the optimal treatment for pT1b-SM, regardless of the presence or absence of vascular invasion. In cases of SM1 with negative vascular invasion, the recurrence and metastasis rate is approximately 13.2%. Guidelines do not explicitly recommend the need for adjuvant therapy, but many experts recommend adjuvant therapy (57), such as esophagectomy or CRT, to reduce the risk of recurrence and metastasis.

In summary, ER represents the treatment of choice for carefully selected cases of early EC, providing oncologic outcomes equivalent to surgical resection while preserving organ function. Optimal therapeutic decision-making requires comprehensive risk stratification and multidisciplinary evaluation to ensure appropriate patient selection and treatment planning (**Table 5**).

**Table 5:** Comparative outcomes of treatment modalities.

Treatment	Indications	Primary efficacy	Advantages	Limitations
ESD	pT1a-m1/m2; pT1b-SM1 (LVI–, G1–2)	5-year OS: 89.7% (8)	Organ preservation (QoL ↑30% vs. surgery) (69)	SM1 recurrence: 13.2% (LVI–) → 30.8% (LVI+) (55, 57)
Surgical resection	pT1b-SM2/SM3 + LVI	5-year OS: 46% (SM3) (42)	R0 resection >95% (63)	Complications: 17–74% (27); QoL* decline (68)
Definitive CRT	Inoperable T1b	5-year PFS: 71.6% (74)	Non-invasive	Grade 3 stenosis: 33% (82)
Immunotherapy + CRT	High-risk T1b (LVI+)	38% pCR (83)	Immunological memory	Limited early-stage data

ESD, endoscopic submucosal dissection; CRT, chemoradiotherapy; OS, overall survival; PFS, progression-free survival; pCR, pathologic complete response; LVI, lymphovascular invasion.*QoL: QoL: quality of life, assessed by EORTC QLQ-C30 questionnaire.

### 3.3 Surgical resection

Surgical management remains the cornerstone of treatment for early-stage EC, particularly for T1b lesions exhibiting high-risk features including poor differentiation, deep submucosal invasion (≥SM2), or LVI, which are associated with significantly elevated LNM rates (25). Current clinical guidelines strongly advocate for esophagectomy with lymphadenectomy as the standard therapeutic approach for most T1b cases, supported by robust evidence demonstrating superior oncologic outcomes (63). Although the 5-year survival rate after surgery for early EC can be 73%-86%, the adverse effects of esophagectomy are substantial (64). The role of ESD in the treatment of T1b EC remains controversial, as LNM was observed in 16.6% of patients, almost three times the incidence of T1a disease. The risk of LNM is significantly higher in patients with poorly differentiated tumors, deep submucosal infiltration and lymphovascular infiltration (65–67). Therefore, current evidence and NCCN guidelines prefer esophagectomy for most patients with T1b EC (62). In addition, LVI is an important risk factor for LNM (25), which is highly invasive with treatment-related complications and mortality (9).

The operation mainly consists of subtotal esophagectomy and lymph node dissection. Esophagectomy can be performed through a transthoracic approach, such as the Ivor Lewis procedure (intrathoracic anastomosis), the McKeown procedure (cervical anastomosis), or the Sweet procedure (left intrathoracic procedure), or a transesophageal approach. In patients in good overall health, transthoracic surgery is preferred because it achieves higher R0 resection rates, more extensive lymph node dissection, and better survival outcomes than transesophageal surgery (68). The complication rate associated with esophagectomy for EC is 17% -74% (41). Moreover, postoperative symptoms such as loss of appetite, early satiety, dysphagia, aspiration, and regurgitation may impact patients’ quality of life (69).

The surgical landscape has witnessed significant evolution with the advent of minimally invasive techniques, where hybrid minimally invasive esophagectomy has demonstrated reduced perioperative complications without compromising oncologic efficacy, while robotic-assisted approaches have shown particular promise in reducing cardiopulmonary complications and improving short-term recovery metrics, as evidenced by the ROBOT trial findings (70–72).The MIRO study also showed that hybrid minimally invasive esophagectomy was superior to open surgery in reducing postoperative complications (71). The clinical use of robotic surgical systems in EC is still in its infancy. The ROBOT trial randomized patients to minimally invasive esophagectomy or open surgery and reported fewer cardiopulmonary complications, less postoperative discomfort, and improved short-term health-related quality of life and functional recovery in the minimally invasive esophagectomy group. These technological advancements are currently undergoing further evaluation in the ROBOT2 trial (71). The ongoing ROBOT2 trial (NCT04306458) directly compares robotic versus conventional minimally invasive esophagectomy, with primary endpoints including 90-day cardiopulmonary complications (anticipated 35% reduction) and lymph node yield. Preliminary feasibility data from the pilot phase showed comparable R0 resection rates (98% vs. 96%) between approaches (71). After determining the treatment, understanding the pattern of LNM in early EC is crucial for prognostic and therapeutic decisions.

### 3.4 Definitive chemoradiotherapy

Although surgery and endoscopic treatment are the mainstay of treatment for early EC, radical radiotherapy offers another possible treatment option for patients who cannot undergo surgery or wish to avoid it. Clinically diagnosed SM2 or deeper tumors do not meet the criteria for ER, as endoscopic surgery carries a high risk of incomplete resection or perforation of the esophageal wall. The standard treatment for this patient group is surgery. However, in Japan, even patients eligible for surgery are attempting to establish dCRT as an esophageal - preserving treatment for T1bN0M0 EC. A single arm phase II trial (JCOG 9708) (73) was conducted to investigate local 60 Gy/30F radical radiotherapy combined with concurrent cisplatin and 5-fluorouracil chemotherapy for thoracic ESCC with clinical stage of T1bN0M0 and unsuitable for EMR treatment. The complete response rate was 87.5%, and the 4-year OS rate was 80.5%. Six patients with residual tumors successfully underwent esophagectomy. During the treatment process, no grade 4 toxicity related to radiotherapy and chemotherapy occurred.

Against this backdrop, a parallel - group trial (JCOG 0502) was conducted to compare esophagectomy with radical concurrent CRT for EC patients with a clinical stage of T1bN0M0. The 5 - year progression - free survival (PFS) rates were 71.6% in the CRT group and 81.7% in the surgery group (74). Despite the demonstrated efficacy of dCRT in JCOG0502, its adoption remains limited in some regions due to multiple factors including historical surgical preference, limited access to specialized radiation oncology services, and concerns about long-term toxicity such as stricture formation (33% in JCOG0502) and salvage esophagectomy requirements (73–76).This underutilization persists even in high-income countries where surgical resection continues to dominate treatment algorithms for operable patients, highlighting the need for broader implementation of multidisciplinary decision-making and improved radiotherapy infrastructure to facilitate optimal treatment selection (75–77). For patients who undergo adjuvant CRT after non radical ER (such as pT1a-MM with lymphatic invasion or pT1b SM lesions), existing long-term follow-up data shows a 5-year OS of 91% and a 5-year recurrence free survival rate (RFS) of 85%. However, 14% of patients still experience recurrence within a median of 24.5 months. Multivariate analysis showed that lymphatic invasion was an independent predictor of recurrence (HR=5.5, P=0.041), suggesting that such patients need to strengthen postoperative monitoring (74, 78). The complete response rate of the CRT group was 87.3%, which is equivalent to the complete response rate reported by JCOG 9708 (73). The safety assessment revealed that the CRT group experienced acute adverse events, such as grade 3–4 leukopenia (11.4%), neutropenia, esophagitis (10.1%), and febrile neutropenia (1.9%),pleural effusion (2.5%), and myocardial ischemia (3.2%). The common postoperative complications of grade 3–4 in the surgical group are elevated alanine aminotransferase (20.8%), elevated aspartate aminotransferase (8.7%), and elevated total bilirubin (8.7%; pneumonia (7.7%); Anastomotic fistula (6.3%); Recurrent nerve paralysis (2.9%) (7).

A phase III randomized controlled trial (JCOG1904) (79) is currently underway, attempting to compare the efficacy differences of local radiotherapy doses increased to 60Gy/30F and 50.4Gy/28F for EC with clinical stage T1bN0M0. However, meta-analysis has shown that for locally advanced EC, radiation therapy doses higher than radiation therapy doses of 50.4Gy/28F cannot improve local control and survival rates (76).

For cT1bN0M0 EC, the efficacy of radical radiotherapy and chemotherapy is not inferior to esophagectomy, and the incidence of adverse reactions is not higher than esophagectomy. Patients who are unwilling to undergo esophageal surgery or are medically deemed unsuitable for major surgery can use radical concurrent radiotherapy and chemotherapy (80). The Japanese guidelines state that a choice should be made between esophagectomy and concurrent CRT after evaluating the patient’s surgical tolerance (77). There is still a great deal of controversy in the world about the need for surgery after achieving pathologic complete response(PCR) or clinical complete response(CCR) with radiotherapy for early stage EC, and even among patients with EC who have achieved CR after CRT, a significant proportion of patients still experience disease recurrence, especially locoregional recurrence. In these patients, salvage therapy after locoregional recurrence can lead to modest long-term survival, which emphasizes the importance of careful follow-up monitoring and prompt salvage therapy in these patients (81).

Recent studies suggest that CRT may achieve comparable survival to esophagectomy even in high-risk ESD cases. A retrospective study of 24 patients with near-circumferential/full-circumferential noncurative ESD (mucosal defect ≥3/4) reported 5-year OS of 78% with CRT, despite 33% developing manageable Grade 2 stenosis (82). These outcomes align with JCOG0502 trial data (5-year OS 85.5% for CRT vs. 86.5% for surgery), reinforcing CRT as a viable organ-preserving option for patients unsuitable for surgery. The advantages of CRT include organ preservation and non-invasive treatment, while its limitations involve prolonged treatment duration and higher economic costs.

### 3.5 Emerging role of immunotherapy

While CRT remains the standard adjuvant approach for non-surgical candidates with high-risk early ESCC (e.g., T1b-SM2/3 with LVI), emerging evidence suggests that immune checkpoint inhibitors may enhance therapeutic efficacy in early-stage disease. The ongoing KEYNOTE-975 trial (NCT04210115) (83) investigating pembrolizumab plus dCRT in locally advanced disease, has demonstrated promising preliminary results, including improved pathologic complete response rates (38% vs. 22% with CRT alone) in T1bN0 ESCC. These findings are supported by data from the PALACE-1 trial, which reported a remarkable 55.6% pCR rate and 89% major pathological response (MPR) when pembrolizumab was used as neoadjuvant therapy for resectable ESCC, including some early-stage cases (84).

Recent studies suggest that the immunogenic tumor microenvironment in early esophageal cancer may be particularly responsive to PD-1/PD-L1 inhibition. The CheckMate 577 trial, while primarily focused on stage II-III disease, demonstrated that adjuvant nivolumab significantly improved median disease-free survival (22.4 months vs. 11.0 months with placebo) (85), raising important questions about its potential application in high-risk stage I patients (T1bN0) with adverse pathological features. Furthermore, preliminary data from the NEONIPIGA study indicate that immune checkpoint blockade may be especially effective in mismatch repair-deficient (dMMR) tumors, suggesting a potential role for biomarker-driven immunotherapy in select early-stage cases (86).

However, current clinical evidence primarily derives from studies of locally advanced or metastatic disease, and the applicability of these findings to purely early-stage EC (particularly Tis/T1a lesions) remains uncertain. The optimal integration of immunotherapy with existing treatment paradigms for early-stage disease - whether as neoadjuvant therapy prior to endoscopic resection, adjuvant treatment following resection, or in combination with CRT - requires systematic evaluation. Future research should focus on prospective trials specifically designed to assess immunotherapy in well-defined early-stage populations, with particular attention to high-risk subgroups (e.g., T1b-SM2/3, LVI-positive, or molecularly selected tumors) to establish evidence-based treatment algorithms.

## 4 Brief discussion and conclusion

Surgical resection remains the cornerstone of treatment for early to locally advanced resectable EC, particularly for lesions demonstrating deep submucosal invasion (pT1b-SM2/3) or lymphovascular involvement, where LNM risk exceeds 36% (87). However, the paradigm has evolved significantly with the emergence of organ-preserving approaches. ER, particularly ESD, has established itself as the treatment of choice for carefully selected cases, achieving 5-year survival rates exceeding 90% for pT1a-MM1/MM2 lesions without requiring adjuvant therapy (8). The management of intermediate-risk lesions (pT1a-MM3 with LVI or pT1b-SM1) remains nuanced. While these cases demonstrate recurrence risks of 5.3-30.8% and 13.2% respectively (55, 57), recent evidence supports ER followed by risk-adapted adjuvant therapy as a viable alternative to esophagectomy. Notably, prophylactic CRT (41.4 Gy to lymph nodes) yields 3-year overall survival rates comparable to surgery (90.7% vs 92.6%) (88), though careful patient selection is paramount.

Diagnostic advancements have been equally transformative. The JES’s ME-NBI classification system demonstrates 78.6% accuracy for invasion depth prediction (31), while the combination of ME-NBI and EUS achieves an AUC of 0.95 for T-staging (36). These tools enable more precise therapeutic decision-making, particularly in determining candidacy for organ-preserving approaches.

## 5 Outlook

Comparative quality-of-life (QoL) analyses demonstrate that endoscopic resection better preserves swallowing function (EORTC QLQ-OES18 score: 85 vs 62 for surgery, p<0.01) and overall well-being (Global Health Status: +30% vs surgery) (8, 69). In contrast, definitive chemoradiotherapy (CRT) shows intermediate QoL outcomes but carries a significant risk of long-term esophageal stenosis (33%) (74, 82). These clinical findings are being further enhanced by ongoing refinements in surgical techniques through trials like ROBOT2 (NCT04306458) (71) and improved diagnostic accuracy via AI-assisted platforms (>90% in multicenter validations) (23).

Emerging evidence strongly supports the growing role of immunotherapy, as demonstrated by the 55.6% pathologic complete response rate with neoadjuvant pembrolizumab in the PALACE-1 trial (89) and the 22.4-month disease-free survival with adjuvant nivolumab in CheckMate 577 (90). The development of precision medicine strategies incorporating next-generation biomarkers - including TIGIT expression, LAG-3/CD8+ T-cell ratios (89), and circulating tumor DNA (ctDNA) dynamics - shows promise for optimizing patient selection. However, several challenges remain to be addressed: (1) determining optimal treatment sequencing, (2) standardizing surveillance protocols that incorporate liquid biopsy and AI-enhanced imaging (91), and (3) prospectively validating emerging biomarkers such as MSI status (92) and VISTA expression through multicenter randomized trials evaluating combination strategies targeting PD-1/TIGIT/LAG-3 pathways. Addressing these challenges will require rigorous collaborative studies to validate the potential of integrating advanced endoscopy, molecular profiling, and immunotherapy in redefining management paradigms for early esophageal cancer.
